# Recent Advances in Understanding the Role of IKKβ in Cardiometabolic Diseases

**DOI:** 10.3389/fcvm.2021.752337

**Published:** 2021-12-08

**Authors:** Rebecca Hernandez, Changcheng Zhou

**Affiliations:** Division of Biomedical Sciences, School of Medicine, University of California, Riverside, Riverside, CA, United States

**Keywords:** cardiometabolic diseases, atherosclerosis, insulin resistance, obesity, IKK-beta, NF-kB

## Abstract

Cardiometabolic diseases, including cardiovascular disease, obesity, and diabetes, are the leading cause of mortality and morbidity worldwide. Cardiometabolic diseases are associated with many overlapping metabolic syndromes such as hypertension, hyperlipidemia, insulin resistance, and central adiposity. However, the underlying causes of cardiometabolic diseases and associated syndromes remain poorly understood. Within the past couple of decades, considerable progresses have been made to understand the role of inflammatory signaling in the pathogenesis of cardiometabolic diseases. The transcription factor, NF-κB, a master regulator of the innate and adaptive immune responses, is highly active in cardiometabolic diseases. IκB kinase β (IKKβ), the predominant catalytic subunit of the IKK complex, is required for canonical activation of NF-κB, and has been implicated as the critical molecular link between inflammation and cardiometabolic diseases. Recent studies have revealed that IKKβ has diverse and unexpected roles in mediating adiposity, insulin sensitivity, glucose homeostasis, vascular function, and atherogenesis through complex mechanisms. IKKβ has been demonstrated as a critical player in the development of cardiometabolic diseases and is implicated as a promising therapeutic target. This review summarizes current knowledge of the functions of IKKβ in mediating the development and progression of cardiometabolic diseases.

## Introduction

Cardiometabolic diseases such as atherosclerosis, obesity, and diabetes are related to several risk factors termed cardiometabolic syndromes (1, 2). Cardiometabolic syndromes encompass a group of metabolic dysfunctions like hypertension, hyperlipidemia, insulin resistance, and central adiposity (1). Chronic low-grade inflammation has been established as a major contributor to the development of cardiometabolic diseases such as type 2 diabetes and atherosclerosis (3, 4). Many inflammatory pathways that contribute to the cardiometabolic disease risk are regulated by the transcriptional factor NF-κB, a master regulator of the innate and adaptive immune responses (1, 5). In non-stimulated cells, NF-κB remains in the cytoplasm bound to specific inhibitory proteins—the inhibitors of NF-κB (IκBs). In response to various stimuli including proinflammatory cytokines, infectious agents, reactive oxygen species, and free fatty acids (FFAs), NF-κB can be rapidly activated through the IκB kinase (IKK) complex (1, 5, 6). The IKK complex is composed of two catalytic subunits (IKKα and IKKβ) and a regulatory subunit (IKKγ/NEMO). Activation of IKK can lead to the phosphorylation and ubiquitination of IκB. Consequently, free NF-κB can then translocate to the nucleus and regulate the expression of many target genes (1, 7).

**Figure 1:**
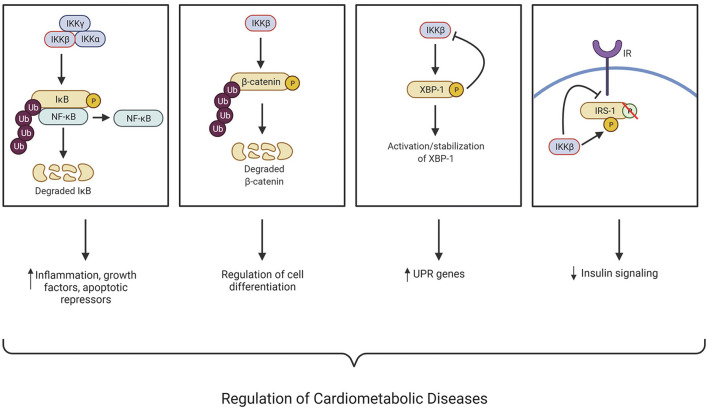
IKKβ regulates many cellular processes associated with the development of cardiometabolic diseases through NF-κB-dependent and -independent mechanisms. IκB kinase (IKK); Nuclear factor kappa B (NF-κB); X-box binding protein (XBP-1); Insulin receptor substate 1 (IRS-1); Insulin receptor (IR); Ubiquitination (Ub); Unfolded protein response (UPR); Phosphorylation (P). This figure was created using BioRender.com.

While IKKβ and IKKα, have a similar structure, they have different functions as IKKα contains a putative nuclear localization signal and IKKβ contains a ubiquitin binding domain. In addition, IKKβ activation is necessary for canonical NF-κB pathway activation, while IKKα is not (8, 9). The stimuli that can activate IKKβ include proinflammatory cytokines, growth factors, microbial products, stress stimuli, and the engagement of T cell receptors. These stimuli can activate membrane-bound receptors such as the Tumor necrosis factor receptor superfamily (TNFRSF), Interleukin-1 receptor (IL-1R), and Toll-like receptors (TLR), subsequently leading to the activation of the IKK complex (10, 11). IKKβ and its serine-threonine kinase activity are essential for regulating inflammatory and immune responses, and many studies have uncovered its function in chronic inflammation-associated cardiometabolic diseases such as atherosclerosis, obesity, and insulin resistance (Figure 1). In addition to regulating the NF-κB pathway, more and more new targets of IKKβ have also been identified. The known IKKβ substrates and their functions in tumorigenesis, inflammation, diabetes, hormone response, and cell survival have been discussed in detail in several comprehensive reviews (12, 13). For the purpose of this review, we focus on IKKβ, its known substrates, and their functions in the development of cardiometabolic diseases (Figure 1).

## The Role of IKKβ in Atherosclerosis Development

Atherosclerosis is the major contributing risk factor for the development of cardiovascular disease (CVD). It is a very complex disease involving the development of plaques in large arteries causing narrowing of the vessel lumen leading to various clinical manifestations, including stroke, ischemic heart disease, chronic kidney disease, and peripheral artery disease. The plaques are characterized by accumulating lipids and immune cells into the sub-endothelial space (14–18). Atherosclerosis has been characterized as a chronic inflammatory disease, which may be initiated when the endothelium undergoes a phenotypic change, termed endothelial dysfunction, stimulated by modified LDL such as oxidized-LDL (oxLDL) and inflammatory stimuli. The endothelium fails to maintain vascular homeostasis during endothelial dysfunction like vasodilation, eliminating reactive oxygen species, and maintaining an appropriate inflammatory balance. Various chemotactic factors and adhesion molecules are differentially expressed by endothelial cells undergoing endothelial dysfunction, which aids in monocyte migration and infiltration. Ox-LDL is rapidly taken up by monocyte scavenger receptors upon monocyte infiltration, leading to the conversion of monocytes into lipid-filled macrophage foam cells. The lesional foam cells can release inflammatory factors to further contribute to the monocyte and lipid build-up (16–19). While acute vessel wall inflammation leads to asymptomatic fatty streaks, chronic inflammation can cause the gradual and uncontrolled accumulation of macrophage foam cells that later develop into symptomatic atheromas or plaques. For many years, the NF-κB pathway has been implicated in the pathogenesis of atherosclerosis (20). For example, NF-κB activation has been detected in atherosclerotic plaques, including macrophages, endothelial cells, and smooth muscle cells in both human and animal models (21–24). Previous studies have implicated that NF-κB activation in human atherosclerosis was IKKβ-dependent and resulted in up-regulation of proinflammatory and prothrombotic mediators (25). However, recently studies have demonstrated that the functions of IKKβ in atherosclerosis are complex and that IKKβ in different tissues or cell types may have different impact on atherosclerosis development in animal models.

### Endothelial Cell IKKβ in Atherosclerosis

As a significant player in atherosclerosis initiation and progression, studies have suggested that the inflammatory response in endothelial dysfunction can be driven by IKKβ/NF-κB signaling (14, 21, 26). Gareus et al. previously demonstrated that inhibition of NF-κB activity through the deletion of IKKγ, also known as NF-κB essential modulator (NEMO), or expression of a dominant-negative IκBα decreases atherosclerosis in atherogenic prone mice (14). They also found that inhibition of NF-κB in endothelial cells reduced the expression of proinflammatory cytokines, chemokines, and adhesion molecules, leading to decreased monocyte recruitment into the plaque (14). Consistently, inhibition of IKKβ in human umbilical vein endothelial cells has been shown to block NF-κB activation, leading to decreased adhesion molecule gene expression including E-selectin, ICAM-1, and VCAM-1 (27). These adhesion molecules are essential for the attachment and infiltration of the recruited monocytes into the intimal layer (16–18). By contrast, constitutive activation of endothelial IKKβ in mice increased monocyte infiltration into the subintimal space, which contributed to exacerbating early and late-stage atherosclerosis (28). Indeed, the rise of age-associated endothelial dysfunction is correlated with increased IKK activation in arteries while pharmacological inhibition of IKK by salicylate has been shown to improve age-related endothelial dysfunction (29). Thus, targeting endothelial cell IKKβ may have beneficial effects against atherosclerosis development.

### Macrophage IKKβ in Atherosclerosis

The M1, or proinflammatory, macrophage plays a key role in atherosclerosis development, while M2, or anti-inflammatory, macrophages enhance plaque regression and stability (30). The link between macrophage polarization and IKKβ remains elusive, though evidence suggests that IKKβ/NF-κB pathway activation polarizes macrophages to the M2, anti-inflammatory phenotype through negative crosstalk with STAT1 (31, 32). To study the role of macrophage IKKβ in atherosclerosis, Kanters et al. transplanted IKKβ-deficient bone marrow-derived macrophages into atherogenic prone low-density lipoprotein receptor-deficient (LDLR^−/−^) mice. They found that the mice receiving IKKβ-deficient macrophages exhibited enhanced atherosclerotic lesion development and increased necrosis, which suggest a protective role of bone marrow-derived macrophage IKKβ against atherosclerosis development (33). However, the same group used a similar method to delete IκBα in myeloid cells, aimed to activate NF-κB signaling. Interestingly, those mice displayed increased atherosclerosis lesion size and leukocyte adhesion without significantly increasing NF-κB targeted genes (34), indicating pro-atherogenic effects of canonical NF-κB activation. Several other studies have also found that macrophage IKKβ/NF-κB pathway has pro-atherogenic effects (35, 36). For example, inhibition of NF-κB in macrophages through the overexpression of a trans-dominant and non-degradable form of IκBα can reduce macrophage foam cell formation *in vitro* (35). Further, myeloid-specific IKKβ deficiency decreased diet-induced atherosclerosis in LDLR^−/−^ mice by diminishing macrophage inflammatory responses such as adhesion, migration and lipid uptake in macrophages (36). Collectively, these results indicate the role of macrophage IKKβ/NF-κB in atherogenesis is complex and more studies are needed to completely understand how IKKβ functions in myeloid cells to regulate atherosclerosis development.

### Vascular Smooth Muscle Cell IKKβ in Atherosclerosis

In addition to endothelial and immune cells, vascular smooth muscle cells (VSMCs) also play an important role in atherogenesis. In the early stages of atherosclerosis, VSMCs undergo a phenotypic switch from contractile to synthetic where they gain the ability to proliferate and migrate into the intimal layer. This provides a beneficial effect as these VSMCs proliferate and migrate to the cap of the plaque and reinforces its stability, lowering the risk for plaque rupture (37). An earlier study demonstrated that IKKα and IKKβ was activated in IL-1β-induced proliferative response of human saphenous vein smooth muscle cells (38). Notably, the proliferative ability of human VSMCs were diminished in IKKα and IKKβ mutant transfected cells (38). The role of VSMC IKKβ in atherosclerosis was also investigated in LDLR^−/−^ mice (39). Deficiency of IKKβ in VSMCs driven by a SM22Cre-IKKβ-flox system protected LDLR^−/−^ mice from diet-induced vascular inflammation and atherosclerosis development (39). Since inhibition of NF-κB activity in endothelia cells also decreased vascular inflammation and atherosclerosis in ApoE^−/−^ mice (14), these studies suggest that inhibiting IKKβ/NF-κB signaling in the vasculature has anti-atherogenic effects.

### Adipocyte IKKβ in Atherosclerosis

Under pathological conditions, adipose tissue is at a chronic low level of inflammation (3). The circulating inflammatory mediators secreted by adipocytes participate in vascular dysfunction, which can lead to atherosclerosis (40). However, the role of adipocyte IKKβ signaling in atherogenesis is poorly understood. A recent study found that adipocyte-specific deletion of IKKβ did not affect obesity and atherosclerosis in lean LDLR^−/−^ mice when fed a low-fat diet (41). When fed a high-fat diet, however, IKKβ-deficient LDLR^−/−^ mice had defective adipose remodeling, leading to increased adipose tissue and systemic inflammation (41). Deficiency of adipocyte IKKβ did not affect atherosclerotic lesion size but resulted in enhanced lesional inflammation and increased plaque vulnerability in obese IKKβ-deficient LDLR^−/−^ mice (41). In addition to regular fat depots, adipocytes can also be found adjacent to the vascular wall called perivascular adipose tissue (PVAT). Under homeostatic conditions, PVAT holds a protective role on vascular homeostasis by secreting bioactive molecules like adiponectin, nitric oxide (NO), and IL-10 (42, 43). However, under pathological conditions, PVAT switches to a proinflammatory phenotype by secreting adipokines, cytokines, and chemokines (43). The role of PVAT in atherosclerosis and vascular injury has not been extensively investigated. However, studies have found that PVAT may contribute to endothelial dysfunction (42), macrophage migration, and VSMC proliferation and migration (44). The role of PVAT IKKβ in vascular function and atherosclerosis remains elusive. Future studies should be considered to investigate the role of PVAT IKKβ/NF-κB signaling on vascular function and atherosclerotic development under normal or pathological conditions (e.g., obesity).

## The Role of IKKβ in Regulating Adiposity

Obesity is a worldwide epidemic and a risk factor for developing severe metabolic and cardiovascular diseases. According to the updated 2020 Heart Disease and Stroke Statistics, 39.6% of adults and 18.5% of youth are living with obesity in the US (45). Thus, research surrounding this field has become increasingly popular due to the financial, economic, and mental burden it carries (46). Obesity is associated with a low-grade chronic inflammation that contributes to the development of many chronic diseases including insulin resistance, diabetes, and CVD (3, 4, 47–49). Adipocytes are responsible for energy storage and respond to overnutrition by increasing adiposity and inflammation. There are three general steps to adipose chronic inflammation. First, adipocytes are introduced to a stressor, like overnutrition. The adaptive physiological response, which includes acute inflammation, aims to balance, and reduce this stressor. However, chronic exposure to this stressor creates new set basal points, which includes higher blood glucose levels and increased body weight (50). Thus, understanding the mechanistic link between inflammatory pathways in obesity, and obesity induced metabolic disorders is critical for developing essential therapeutic targets.

The IKKβ/NF-κB pathway is highly active in the adipose tissues of obese patients and in mouse models of obesity and insulin resistance (1, 51, 52). In addition to regulating inflammatory responses, IKKβ also plays important roles in regulating cell proliferation, differentiation, survival, and apoptosis (47, 53). However, the function of IKKβ during obesity in the context of adipose tissue development remain elusive. Recent studies have revealed the previously unrecognized function of IKKβ in regulating adiposity.

### Adipocyte Progenitor IKKβ in Regulating Adiposity

While deletion of IKKβ in VSMCs decreased atherosclerosis development in LDLR^−/−^ mice (39), those mice were also protected from diet-induced obesity and insulin resistance. Interestingly, many adipocyte precursor cells express SMC markers and ablation of IKKβ blocked adipocyte differentiation *in vitro* and *in vivo*, suggesting that IKKβ functions in adipocyte precursor cells to regulate adiposity (39). Indeed, selective deletion of IKKβ in the white adipose lineage further elucidated the role of adipose progenitor cell IKKβ signaling in regulating adiposity and metabolic function (39, 54). Deficiency of adipose progenitor IKKβ decreased high-fat feeding-induced adipogenesis and systemic inflammation, resulting in decreased adiposity and insulin resistance in those mice (39, 54). The function of IKKβ in the regulation of adipogenesis was further confirmed in mesenchymal stem cells (MSCs) (55). Mechanistic studies then revealed an important crosstalk between IKKβ and Wnt/β-catenin signaling (Figure 1) (55). Interestingly, IKKβ is a β-catenin kinase that can directly phosphorylate the conserved degron motif of β-catenin to prime it for β-TrCP-mediated ubiquitination and degradation (10, 55). Wnt/β-catenin signaling has been well studied to inhibit adipocyte differentiation (56, 57) and the impact of IKKβ signaling on adipogenesis was abolished in β-catenin-deficient MSCs (10, 55). Thus, IKKβ-mediated β-catenin phosphorylation may play a critical role in regulating adipocyte differentiation and adiposity in obesity (Figure 1).

### Adipocyte IKKβ in Regulating Adiposity

While studies have suggested a pro-obesogenic role of progenitor IKKβ, the function of IKKβ in mature adipocytes is apparently more complicated. Constitutive activation of IKKβ in adipocytes has been demonstrated to increased energy expenditure in mice, leading to protective effects against diet-induced obesity and insulin resistance (58). However, targeted deletion of IKKβ in adipocytes did not affect obesity but resulted in increased tissue inflammation, impaired adipose remodeling, and exacerbated metabolic disorders (59, 60). In addition to mediating inflammation, IKKβ can also promote cell survival by upregulating NF-κB-mediated anti-apoptotic gene expression (61–63) and by direct phosphorylation of pro-apoptotic protein, BAD (64). Previous reports have linked adipocyte death with obesity, adipocyte macrophage infiltration, and systemic insulin resistance (65). IKKβ has been shown to be a key adipocyte survival factor in obesity, and deficiency of IKKβ in adipocytes can lead to high fat feeding-elicited cell death, impaired adipose tissue remodeling and partial lipodystrophy in visceral adipose tissue (59, 60). Further studies are required to completely understand the role of adipocyte IKKβ in regulating energy expenditure, homeostasis, and adiposity.

## The Role of IKKβ in Insulin Resistance

Insulin resistance is a very complex syndrome and IKKβ has been shown to regulate insulin resistance by directly interfering with the insulin signaling pathway (66). Once stimulated by its ligand, insulin, the insulin receptor (IR) becomes activated and phosphorylates insulin receptor substrate-1 (IRS-1) on its tyrosine residues, leading to increased glucose uptake (67). As a serine kinase, IKKβ can ectopically phosphorylate IRS-1 on multiple serine residues, which impairs insulin signaling (Figure 1) (68). Several studies have demonstrated that treatment with glucose lowering drugs and molecules such as kaempferol (69), timosaponin B-II (TB-II) (70), rosiglitazone (71), and bovine α-lactalbumin hydrolysates (α-LAH) (72) can alleviate insulin resistance by decreasing or inhibiting IKKβ levels/activity resulting in a reduction of ectopic IRS-1 serine phosphorylation.

### Hepatic IKKβ in Insulin Resistance

The IKKβ/NF-κB pathway has been demonstrated to be active in both obesity-dependent and independent insulin resistance (47, 53). Inhibition of IKKβ with salicylate or other methods is associated with reduced insulin resistance and glucose intolerance (54, 73–75). Previous studies demonstrated that constitutively active hepatic IKKβ induced obesity-independent systemic insulin resistance, while inhibiting hepatic NF-κB reversed both local and systemic insulin resistance (51, 76). These findings indicate an important role of IKKβ in regulating hepatic and systemic insulin resistance. Another study utilizing hepatocyte-specific IKKβ deficient mice found improved hepatic insulin response while maintaining systemic insulin resistance during obesity (77). These results can be attributed to obesity-associated systemic inflammation that cannot be alleviated by IKKβ knockdown in the liver alone. More recently, it has been reported that hepatic IKKβ in the liver can improve glucose homeostasis by interacting with x-box binding protein 1 (XBP1) and enhancing its activity, stabilization, and nuclear translocation (Figure 1) (78). While it is generally recognized that hepatic inflammation drives the detrimental perspectives of obesity-induced insulin resistance (1, 73, 79), upregulation of certain inflammatory signaling could have positive or negative contributions to whole-body metabolism, depending on conditions of signaling activation and related physiological statuses. Therefore, the hepatic IKKβ function in insulin resistance is complex and future studies are required to define the detailed mechanisms through which hepatic IKKβ regulates insulin responsiveness under normal and pathophysiological conditions.

### Adipose IKKβ in Insulin Resistance

Inflammation is an important contributor of insulin resistance, and adipose tissue is one of the important tissues for this high-fat feeding-elicited inflammatory response (80). Adipose IKKβ signaling has been implicated in obesity-associated insulin resistance. For example, studies have found that IKKβ deficiency in adipocyte precursors or adipose lineage cells can protect mice from diet-induced obesity, systemic inflammation and insulin resistance (39, 54). Several studies demonstrated that IKKβ deficiency and XBP1 overexpression attenuates FFA-induced inflammation and impairment of insulin signaling in cultured adipocytes (81, 82). While hepatic IKKβ increases nuclear translocation of XBP1 (78), adipocyte IKKβ is inhibited by XBP1 (82), indicating a more complex role of IKKβ/XBP1 interaction in cardiometabolic disease. Overexpression of IKKβ in adipocytes also led to increased adipose tissue inflammation in mice (58). Paradoxically, those mice were resistant to diet-induced obesity and insulin resistance, likely due to increased energy expenditure (58). Deletion of adipocyte IKKβ did not affect obesity in mice but resulted in elevated adipose tissue inflammation, increased macrophage infiltration and exacerbate insulin resistance (59, 60).

### Skeletal Muscle IKKβ in Insulin Resistance

Skeletal muscle is another insulin responsive tissue that is impaired in obesity and diabetes (67). Studies revealed elevated IKKβ activity in isolated skeletal muscle of obese patients with type 2 diabetes and obese mice (83, 84). By contrast, inhibition of IKKβ or NF-κB signaling can restore insulin signaling *in vitro* (85, 86) and systemic IKKβ inhibition can alleviate skeletal muscle and systemic insulin resistance all together (73, 74). However, under obese conditions, targeting skeletal muscle IKKβ can only alleviate local insulin resistance, but not systemic insulin responsiveness (87).

### Myeloid IKKβ in Insulin Resistance

While tissue-specific inhibition of IKKβ (i.e., liver, adipose, skeletal muscle) may be able to abrogate local insulin resistance, it may not be sufficient for systemic inflammation-induced insulin resistance under obese conditions. For example, it is reported that myeloid-specific IKKβ deficiency can improve obese-dependent systemic insulin resistance (77, 87), indicating that myeloid cell IKKβ plays a role in systemic insulin resistance and inflammation in obesity. Furthermore, Cai et al. linked the IKKβ/NF-κB pathway with paracrine IL-6 signaling (51), which is associated with type 2 diabetes and insulin resistance (88, 89). IL-6 can induce the expression of suppressor of cytokine signaling 3 (SOCS-3), which inhibits autophosphorylation of IRS-1 and insulin receptor (90). The IKKβ/NF-κB/IL-6 axis was confirmed to be involved in insulin resistance when IL-6 neutralization improved insulin resistance (51).

## The Role of IKKβ in Metabolic Syndrome-Associated Liver Disease

Non-alcoholic fatty liver disease or non-alcoholic steatohepatitis is also associated with metabolic syndrome. The activation of the IKKβ/NF-κB pathway has been shown to promote fatty liver disease, or hepatic steatosis (91), whereas inhibition of IKKβ prevents the initiation of steatosis and non-alcoholic steatohepatitis (75, 92). Inhibition of IKKβ significantly reduced the expression of essential proinflammatory genes like TNFα and IL-6 in the liver (92). In line with lipid metabolism, the peroxisome proliferator-activated receptor family (PPAR) is an important regulator of lipid homeostasis in multiple organs and tissues (93). PPARα, highly expressed in the liver, can upregulate IκB, thus inhibiting the NF-κB pathway (92, 93). Interestingly, IKKβ inhibition can also lead to PPARα upregulation and reduced lipid accumulation in the liver by increasing CPT-1 and ACOX—two important molecules that decreases fatty acid accumulation through β-oxidation (92). Additionally, IKKβ inhibition attenuated hepatic inflammation, apoptosis, and collagen deposition, therefore preventing liver fibrosis (54, 92). By contrast, hepatic IKKβ activation promoted liver fibrosis by inducing chronic inflammation (94). While the mechanism behind IKKβ-mediated hepatic steatosis and fibrosis remain to be explored, these findings suggest that inhibiting IKKβ may prevent lipid and collagen accumulation in the liver, leading to decreased hepatic steatosis and fibrosis development.

## The Role of IKKβ of the Central Nervous System in Cardiometabolic Diseases

### IKKβ of the Central Nervous System in Obesity and Insulin Resistance

Although there have been strong links between IKKβ and metabolic diseases within the periphery, more recently, inflammatory activation has been seen within the central nervous system (CNS). Specifically, IKKβ in the hypothalamus can be activated in obesity and obesity-related metabolic dysregulation such as energy, body weight, and glucose dysregulation (95–98). A study found that FFAs induce TLR4-mediated hypothalamic cytokine production and anorexigenic signal resistance which may lead to obesity (99). Signaling between the gut and brain (gut-brain-axis) is a major influencer in developing obesity. Obese mice and mice stimulated with overnutrition display overall higher levels of IKKβ within the hypothalamic neurons, which is consistent with the systemic trend (95, 100). However, it was observed that overnutrition-mediated activation of IKKβ/NF-κB was activated intracellularly by ER stress and prompted both hypothalamic leptin and insulin resistance through the induction of suppressor of cytokine signaling 3 (SOCS3), an inhibitor of leptin and insulin signaling (95, 101). ER stress can also lead to impaired hepatic insulin signaling, which was improved upon ER stress inhibition (102). TLR-dependent IKKβ activation in the CNS was also involved in obesity and leptin resistance (96). Deficiency of IKKβ in hypothalamic AGRP neurons displayed anti-obese phenotype along with preserved leptin and insulin signaling and reduced SOCS3 gene expression, and overexpression of SOCS3 reversed the protective effects of IKKβ knockout in mice (95). By contrast, activation of IKKβ in AGRP neurons resulted in impaired glucose homeostasis, without affecting body weight and leptin signaling (103).

While it is critical to study the effects of hypothalamic inflammation on obesity and metabolic syndromes, it is also important to investigate the upstream targets mediating hypothalamic inflammation. For example, astrocytes play essential roles in neuronal development; regulation of blood flow; fluid, ion, pH, and transmitter homeostasis; the regulation of synaptic transmission; and regulate immune response (104). Under pathological conditions or external stressors, astrocytes and other glial cells undergo gliosis, or astrogliosis, which is characterized by proliferation and accumulation of astrocytes (104, 105). Zhang et al. demonstrated an important role of astrocyte IKKβ in stimulating glucose intolerance, hypertension, and weight gain (106). While overnutrition and IKKβ overexpression inhibited proper astrocytic plasticity, inhibition of IKKβ prevented overnutrition-induced metabolic diseases and impaired astrocytic plasticity (106). Mechanistically, IKKβ-induced shortening of astrocyte processes led to increased extracellular GABA, an inhibitory neurotransmitter, and lower brain derived neurotrophic factor (BDNF) levels through inhibition of BDNF secreting neurons in the hypothalamus (106). Low levels of BDNF have been associated with metabolic disorders such as obesity, energy metabolism, and hyperglycemia (107). The protective role of IKKβ deficiency in astrocytes were reversed by BDNF inhibition, suggesting that the GABA-BDNF axis is important in regulating energy homeostasis and metabolic syndromes (106). In addition to developed cells within the CNS, the hypothalamic neural stem cells are important mediators for metabolic syndrome. IKKβ/NF-κB activation in the mediobasal hypothalamus can lead to obesity and insulin resistance, along with loss of neuronal development including POMC neurons (108).

### IKKβ of the Central Nervous System in Hypertension

Hypertension, a chronic elevation in arterial blood pressure, is one of the major risk factors for developing CVD such as myocardial infarction, stroke, and heart failure. Although there are therapeutic interventions aimed to target and treat hypertension, it is still a prevalent contributor to cardiometabolic disease burden (109). IKKβ in the CNS, mainly in the hypothalamus, can regulate blood pressure. Overexpression of a constitutively active form of IKKβ in the mediobasal hypothalamus induces hypertension in mice, while NF-κB inhibition attenuated high-fat feeding induced hypertension in mice (110). Additionally, astrocyte-specific IKKβ overexpression in mice led to higher daytime blood pressure, while NF-κB inhibition reversed obesity-induced hypertension in mice (106). In line with the previous discussion linking ER stress to insulin resistance, thapsigargin-induced ER stress increased blood pressure and phosphorylated IκB, but inhibition of NF-κB alleviated these effects (102).

## Conclusion

Recent research advancements have expanded our knowledge on the function of IKKβ in cardiometabolic diseases. A summary of the role of IKKβ in cardiometabolic diseases is listed in Table 1. By exploring various mechanisms of chronic inflammation-associated diseases, such atherosclerosis, obesity, and insulin resistance, IKKβ and its regulated main canonical NF-κB pathway in various cell types have been found to play diverse roles in cardiometabolic disease development. In addition, new discoveries revealed that NF-κB-independent mechanisms may also contribute to the impact of IKKβ on the development of cardiometabolic diseases. For example, IKKβ can interact with several important signaling molecules such as β-catenin, BAD, and IRS-1 that are essential for regulating cell survival, differentiation and insulin signaling. With more new molecular targets of IKKβ being discovered, there will be more opportunities for fully understanding the complex function of IKKβ in cardiometabolic diseases and for developing new and effective therapeutic approaches.

**Table 1:** Overview of IKKβ modulation and mechanism in cardiometabolic diseases.

**Cell Type**	**IKKβ modulation**	**Effect on cardiometabolic diseases**	**Mechanism**	**Reference**
Endothelial Cells	Constitutive activation	Accelerated atherosclerotic development and progression, increased macrophage infiltration	1. Upregulation of endothelial NF-κB mediated gene expression of cytokines/chemokines (CCL2, CCL12, IL-1β, IL-6, CXCR4), increased macrophage infiltration 2. Cellular transition of SMC to macrophage-like cells	(29)
Myeloid Cells	Knockout	Increased lesion size, more severe lesion, increased necrosis, increase macrophage content at the lesion site	1. Reduction of IL-10 anti-inflammatory cytokine	(33)
Myeloid Cells	Knockout	Decreased lesion size, macrophage infiltration, and foam cell formation	1. Reduction in macrophage/lesional NF-κB-mediated proinflammatory gene expression/protein level (MCP-1, TNFα, IL-1β, IL-1α, VCAM-1, ICAM-1), reducing macrophage recruitment and infiltration 2. Reduced scavenger receptor expression levels, decreased ox-LDL uptake by macrophages	(36)
VSMC	Knockout	Decreased lesion size	1. Reduction in lesion proinflammatory protein level (MCP-1, TNFα, IL-1β)	(39)
Adipocytes	Knockout	Increased plaque vulnerability	1. Upregulation of aortic/lesional NF-κB mediated gene expression of cytokines/chemokines/protein levels (MCP-1, TNFα, IL-1β, IL-6, VCAM-1, ICAM-1)	(41)
MSC	Gain of function	Promoted adipogenesis and inhibits osteogenesis	1. Increases adipogenic genes (Zfp423, PPARγ) 2.Tags β-catenin for β-TrCP-mediated ubiquitination leading to adipogenesis	(55)
MSC, MEFs	Knockdown with various methods	Inhibited adipogenesis and promotes osteogenesis	1. Suppresses adipogenic genes (Zfp423, PPARγ) 2. Reduced β-catenin ubiquitination leading to osteogenesis	(55)
White adipose lineage	Knockout	Decreased obesity; improved glucose tolerance; protected from hepatic steatosis	1. Suppresses adipogenic genes (Zfp423, PPARγ, C/EBPα) 2. Decreases Smurf2 levels resulting in increased β-catenin activity 3. Reduced macrophage infiltration in WAT 4. Decrease in hepatic lipogenic genes (SREBP1c, ScD-1, PPARγ)	(39, 54)
Human stem cells	Pharmacological inhibition	Inhibited adipogenesis	1. Suppresses adipogenic genes (Zfp423, PPARγ, C/EBPα) 2. Decreases Smurf2 levels resulting in increased β-catenin activity	(54)
Adipocytes	Knockout	Increased adipocyte death; macrophage infiltration; defective adipose remodeling; impaired insulin signaling	1. Increases pro-apoptotic genes (XIAP, Bcl2) 2. Activation of proapoptotic protein BAD 3. Increases adipose lipolysis 4. Increase in WAT proinflammatory genes (TNFα, MCP-1, IL-2)	(59)
Hypothalamic AGRP neurons	Knockout	Anti-obese phenotype; reduced glucose intolerance; preserved insulin and leptin signaling	1. Reduction of SOCS3	(95)
Mediobasal Hypothalamus	Constitutive activation	Impaired central insulin and leptin signaling	1. Decreased Akt and PIP3 activation 2. Increased SOCS3	(95)
Systemic	Pharmacological inhibition	Reduced high sucrose diet (HSD)-induced obesity; prevented hepatic steatosis and NASH	1. Reduced WAT inflammation (TNFα, F4/80) 2. Reduced NF-κB-mediated liver inflammation 3. Upregulation of PPARα and PPARγ leading to increased β-oxidation (CPT-1 and ACOX)	(92)
Adipocytes	Constitutive activation	Decreased lipid deposits into other tissue (i.e., hepatosteaotosis); improved systemic insulin resistance	1. Increased energy expenditure through hypothesized mechanisms: increased thermogenesis and fatty acid oxidation (upregulation of CPT-1β, ACO1), increase in mitochondria biogenesis (upregulation of NRF1), elevated IL-6 levels 2. Decreased body weight and systemic inflammation	(58)
Hepatocytes	Knockout	Improved hepatic insulin resistance, sustained peripheral insulin resistance	1. Decrease in proinflammatory gene expression (IL-6) in liver	(77)
Myocytes	Knockout	Retained systemic insulin resistance	1. Maintained high TNFα expression in WAT; low IR activation	(87)
Myeloid cells	Knockout	Improved systemic insulin resistance	1. Decrease in proinflammatory gene expression (IL-6)	(77)
Hepatocytes	Constitutive activeation	Increased liver and peripheral insulin resistance	1. Increased expression of circulating IL-6	(51)
Hepatocytes	Overexpression	Improved insulin sensitivity; improved glucose homeostasis	1. Increased XBP1 stability/decreased XBP1 degradation via IKKβ mediated phosphorylation	(78)
Astrocytes	Overexpression	Induced metabolic syndromes	1. Decreased astrocyte plasticity leading to increased GABA and increased GABA inhibition of BDNF secreting neurons	(106)
Mediobasal Hypothalamus	Activation	Increased obesity and insulin resistance	1. Loss of neuronal development	(108)
Hypothalamic AGRP neurons	Activation	Impaired glucose homeostasis; no change in body weight or leptin signaling	1. Increased AGRP firing	(103)
Systemic	Pharmacological inhibition	Alleviated insulin resistance	1. Reduction of ectopic IRS-1 serine phosphorylation 2. Restoration of IRS-1 phosphorylation and protein levels 3. Enhanced Akt activity 4. Increased glucose uptake 5. Increased glycolysis and glycogen/lipid synthesis	(54, 68–74)
Adipocyte	Knockout	Worsened insulin resistance; enhanced inflammation	1. Reduction of IL-13	(60)
Hepatocytes	Constitutive activation	Increased liver fibrosis	1. Increased inflammation (chemokines) and macrophage infiltration in the liver	(94)

## Author Contributions

RH: conceptualized, wrote, and edited the manuscript. CZ: reviewed, edited, and revised the manuscript. All authors contributed to the article and approved the submitted version.

## Funding

This work was supported in part by National Institutes of Health grants (R01HL131925 and R01ES023470) and American Heart Association grant (19TPA34890065) to CZ. RH was supported by an NIH T32 training grant (T32ES018827).

## Conflict of Interest

The authors declare that the research was conducted in the absence of any commercial or financial relationships that could be construed as a potential conflict of interest.

## Publisher's Note

All claims expressed in this article are solely those of the authors and do not necessarily represent those of their affiliated organizations, or those of the publisher, the editors and the reviewers. Any product that may be evaluated in this article, or claim that may be made by its manufacturer, is not guaranteed or endorsed by the publisher.
